# Supplementary material for: A scoping review protocol of the lived experiences of doing a PhD in Africa
Source: PLoS One. 2023 Sep 6;18(9):e0290701. doi: 10.1371/journal.pone.0290701 (PMC10482273; doi:10.1371/journal.pone.0290701)
Supplement: Attachment — Submitted filename: Response to Reviewers.docx [file pone.0290701.s002.docx]

**Response to reviewer’s file**

| 1. Line 94 – 100 is part of the introduction as a justification for the study method chosen. | This Section has now been joined with the introduction so that it flows better. The method section has been moved to the protocol design as this best represent how the protocol will be undertaken. |
| --- | --- |
| 2. The search strategy referred to as “Step 2 Identifying relevant studies” refers only to health-related databases. For example, utilising MEDLINE and MeSH to generate “key words, scientific terms and medical subject headings” for use in other database searches. If the scoping review is limited to medical/health related PhD candidate experiences only, this needs to be stated clearly in the study title, research question and introduction section. | Other databases have now been included and MeSH will not be used so that articles that capture experiences of participants from other areas will be included. |
| 3. In the search strategy using the broad terms “experience” or “attitude” singly is not efficient. | Phrases have been added so that the search will be more efficient. |
| 4. The search should consider using individual country names (54) not limited to “Africa”. | This has now been addressed so that West, East, South and North Africa have been included in the search terms. Nigeria was specifically included because Nigeria has been reported to have a significant amount of PhD candidates compared to other African Countries |
| 5. The Boolean operator approach should be cited as the method described for combining key words and synonyms. | This has been addressed as well. |
| 6. Describe the process of how duplicates will be identified and removed. | All the articles will be uploaded into Covidence a software that is used to screen references and extract data. This software automatically removes any duplicates that are found within the collection of articles. This has been addressed in step 3: study selection. |
| 7. Describe how reference lists in identified articles will be further investigated. | This has been addressed in step 2. |
| 8. As a scoping review the following objectives may also be considered:  a) The types of methods used in the identified studies and gaps  b) The definitions of terms and description of concepts used in the identified articles  c) Can the study be used as a precursor to inform a systemic review? | This have been included in the objectives and aims of the scoping review. |
| 9. Under study limitations use of published literature only (not grey literature where several other types of evidence on the topic maybe available such as policy reports, national development plan reviews etc). | This has been removed so that all other relevant information will not be missed. |
| 10. Also language limitations (Selecting for English only literature likely to excludes a large proportion of the literature). | This has been addressed. |
| Reviewer #2: Author need to reconcile the title with the main thrust in the article. It would seem that author wish to "document lived experiences" rather than "understand...lived experiences". | The title has been changed so that it represents the aims of the review. |
| Line 36: author needs to clarify if Africa has equally been disadvantaged as subject of research. Author need to explain what is "brain drain" (line 68). | Yes, this is an important point and has now been discussed. |
| Line 78 – 79 clarify the decade in focus. There is lack of consistency between the introduction and the aim of study. The scope and boundaries of the scoping review needs to be better defined. | This is from the 2000s, this has now been clarified. |
| Line 194 - should be supported with evidence in form of literature citation. 206 indicate that research was already conducted while mode of writing shows research "scoping" is to be done. Need to reconcile. Authors need to show better relationship between the PRISMA flow chart and the focus of study. | This statement has now been supported with research. This has been reworded to reflect what will be performed for the scoping review in the future. |
